# Modified Lipoprotein-Derived Lipid Particles Accumulate in Human Stenotic Aortic Valves

**DOI:** 10.1371/journal.pone.0065810

**Published:** 2013-06-07

**Authors:** Satu Lehti, Reijo Käkelä, Sohvi Hörkkö, Outi Kummu, Satu Helske-Suihko, Markku Kupari, Kalervo Werkkala, Petri T. Kovanen, Katariina Öörni

**Affiliations:** 1 Wihuri Research Institute, Helsinki, Finland; 2 Department of Biosciences, University of Helsinki, Helsinki, Finland; 3 Division of Cardiology, Helsinki University Central Hospital, Helsinki, Finland; 4 Division of Cardiothoracic Surgery, Helsinki University Central Hospital, Helsinki, Finland; 5 Institute of Diagnostics, Department of Medical Microbiology and Immunology, University of Oulu, Oulu, Finland; 6 Clinical Research Center, Oulu University Hospital, Oulu, Finland; 7 NordLab Oulu, Oulu University Hospital, Oulu, Finland; University of Padova, Italy

## Abstract

In aortic stenosis plasma lipoprotein-derived lipids accumulate in aortic valves. Here, we first compared the lipid compositions of stenotic aortic valves and atherosclerotic plaque cores. Both pathological tissues were found to be enriched in cholesteryl linoleate, a marker of extracellularly accumulated lipoproteins. In addition, a large proportion of the phospholipids were found to contain arachidonic acid, the common precursor of a number of proinflammatory lipid mediators. Next, we isolated and characterized extracellular lipid particles from human stenotic and non-stenotic control valves, and compared them to plasma lipoproteins from the same subjects. The extracellular valvular lipid particles were isolated from 15 stenotic and 14 non-stenotic aortic valves. Significantly more apoB-100-containing lipid particles were found in the stenotic than in the non-stenotic valves. The majority of the lipid particles isolated from the non-stenotic valves had sizes (23±6.2 nm in diameter) similar to those of plasma low density lipoprotein (LDL) (22±1.5 nm), while the lipid particles from stenotic valves were not of uniform size, their sizes ranging from 18 to more than 500 nm. The lipid particles showed signs of oxidative modifications, and when compared to isolated plasma LDL particles, the lipid particles isolated from the stenotic valves had a higher sphingomyelin/phosphatidylcholine –ratio, and also higher contents of lysophosphatidylcholine and unesterified cholesterol. The findings of the present study reveal, for the first time, that in stenotic human aortic valves, infiltrated plasma lipoproteins have undergone oxidative and lipolytic modifications, and become fused and aggregated. The generated large lipid particles may contribute to the pathogenesis of human aortic stenosis.

## Introduction

The non-rheumatic calcific aortic valve disease, or simply, aortic stenosis (AS), is an active fibrocalcific condition of the aortic valves, and it develops gradually over decades before becoming clinically manifested [1]. The development of AS has been paralleled to the development of atherosclerosis and the two diseases share several risk factors, among them elevated plasma low density lipoprotein (LDL) cholesterol and triglyceride levels [2], [3], [4]. However, there are significant differences in the development of the two diseases and the role of lipid accumulation in AS has been debated, particularly since plasma lipid-lowering therapies have been unsuccessful in slowing AS progression [5], [6].

The lesions in aortic valves emerge subendothelially on the aortic side of the valvular leaflets, where the endothelial cells most likely get damaged as a result of severe shear stress, the dysfunctional endothelium then providing access for plasma lipoproteins and leukocytes to the subendothelial fibrosa layer [7], [8]. Importantly, this subendothelial layer contains a proteoglycan-rich extracellular matrix [9], *i.e.* a matrix offering a suitable ground for lipoprotein retention [10], [11]. Indeed, in aortic valves, apoB-100 has been detected already in early stages of the disease development and its amount increases with the progression of the disease [12], [13].

During progression of the AS, inflammatory cells such as macrophages, T cells, and mast cells accumulate in the diseased valves [14], [15], where the cells can secrete various enzymes and agents capable of modifying the retained lipoproteins. The modified lipoproteins can aggregate and fuse [16] and, in fact, extracellular aggregated and fused lipid particles have been shown to accumulate in the stenotic aortic valves of hypercholesterolemic rabbits [17],[18]. Interestingly, in this animal model, the first sign of lipid accumulation in aortic valves is the appearance of extracellular lipid particles rich in unesterified cholesterol (UC) [17]. The modified lipoproteins can activate the valvular inflammatory cells, which, by secreting proinflammatory cytokines, may further promote the progression of AS by accelerating valvular fibrosis and calcification. Indeed, in stenotic leaflets valvular cells undergo phenotypic transdifferentiation into bone-forming cells, a process that is induced by inflammatory cytokines and oxidized cholesterol [3].

Based on the above information, we hypothesized that the infiltrated lipoproteins undergo extracellular modification, thereby leading to aggregation and fusion of lipoproteins and extracellular lipid accumulation in diseased human aortic valves. To test this hypothesis, we isolated and characterized extracellular lipid particles derived from stenotic aortic valves obtained from patients undergoing valvular surgery. We found that the particles contained apoB-100 and were aggregated and fused having sizes ranging from 18 to 500 nm. When compared to plasma lipoproteins obtained from the same patients, the particles were not only larger, but also showed signs of oxidation and had a decreased phosphatidylcholine/sphingomyelin (PC/SM) –ratio, an increased content of lysophosphatidylcholine (LPC), and an increased ratio of unesterified/esterified cholesterol (UC/CE), all of which suggest that significant modification of the apoB-100-containing lipoproteins had taken place in the stenotic aortic valves during disease progression.

## Materials and Methods

### Ethics statement

The use of human material conforms to the principles outlined in the Declaration of Helsinki and the study was approved by the Ethics Committee of Helsinki University Central Hospital and the National Authority for Medicolegal Affairs. All surgical participants signed an informed consent document.

Human plasma was obtained from healthy blood donors, who had signed an informed consent document. The plasma samples were by-products from the preparation of blood products for clinical use. The use of plasma samples in lipoprotein isolation was approved by the Finnish Red Cross Blood Service.

### Biological samples

Non-rheumatic stenotic aortic valves were obtained from 23 patients (Table 1) undergoing a valve replacement surgery. The control valves (Table 2) were obtained from autopsy samples (n = 3), from patients undergoing a valve replacement surgery due to aortic insufficiency (n = 6), and from heart transplant recipients or donors (n = 5). The diagnostic criteria for AS were defined according to the American College of Cardiology and the American Heart Association 2008 guidelines [19] and were as follows: for severe AS the aortic valve area <1 cm^2^, the mean pressure gradient >40 mmHg, and the aortic jet velocity >4.0 m/s; and for moderate AS the aortic valve area 1–1.5 cm^2^, the mean pressure gradient 25–40 mmHg, and the aortic jet velocity 3–4 m/s. Detailed descriptions of the patients are shown in Tables 1 and 2. Samples of atherosclerotic abdominal aortae were obtained at autopsy (n = 3).

**Table 1:** Clinical characteristics of the patients with aortic valve stenosis.

Subject	Sex	Age. y	BMI	Diagnosis	Clinical history	Statin	Smoking	Dys-lipidemia	Valve leaflet weight (mg)
**A**	F	67	29	AS	Diabetes	**+**	**−**	**+**	1892
**B**	F	82	22	AS+AI	Hypertension, diabetes	**−**	**−**	**+**	633
**C**	M	73	23	AS	Diabetes	**+**	**+**	**+**	605
**D**	F	70	26	AS	-	**−**	**−**	**−**	1773
**E**	M	81	24	AS	Hypertension, kidney disease	**−**	**−**	**−**	2067
**F**	M	74	N/A	AS	Hypertension	**+**	**−**	**+**	930
**G**	M	58	28	AS	Hypertension, diabetes, TIA/stroke	**+**	**+**	**+**	1876
**H**	F	75	30	AS	**-**	**−**	**−**	**+**	1476
**I**	M	80	22	AS	Hypertension	**+**	**+**	**+**	1393
**J**	F	61	23	AS	**-**	**−**	**+**	**−**	1520
**K**	F	70	31	AS	TIA/stroke	**+**	**−**	**+**	659 and 767
**L**	M	53	22	AS	**-**	**+**	**+**	**+**	1950 and 1080
**M**	M	53	27	AS	Hypertension	**+**	**−**	**+**	1827 and 2367
**N**	M	64	37	AS	Diabetes	**−**	**−**	**+**	1668
**O**	F	86	N/A	AS	**-**	**−**	**−**	N/A	852
**P**	F	72	21	AS	Hypertension	**+**	**−**	**+**	779
**Q**	M	43	37	AS	-	**+**	**−**	**+**	4367
**R**	M	37	27	AS+AI	Hypertension	**−**	**+**	**+**	295
**S**	F	86	22	AS+AI	-	**−**	**−**	**−**	2308
**T**	F	75	30	AS	-	**−**	**−**	**−**	392
**U**	F	65	28	AS+AI	-	−	−	N/A	778
**V**	F	76	33	AS	Hypertension, Diabetes	+	−	+	1321
**X**	M	63	31	AS	Hypertension	+	+	+	1594

AS indicates aortic stenosis; AI, aortic insufficiency; N/A, data not available. Aortic valve leaflets of patients A–O were used for characterization of the size and composition of the extracellular particles. Extracellular particles isolated from the valve leaflets of patients P and Q, and from additional leaflets of patients K, L and M were assayed for oxidative modification. Valve leaflets of patients R–X were used for lipid extraction and lipidomic analysis by mass spectrometry.

**Table 2:** Clinical characteristics of the patients without aortic valve stenosis, whose aortic valve leaflets were used as controls.

Subject	Sex	Age. y	BMI	Diagnosis	Clinical history	Statin	Smoking	Dyslipidemia	Valve leaflet weight (mg)
**a**	M	69	25	AI	Hypertension.	**−**	**−**	**−**	214
**b**	M	65	30	AI	Kidney disease, Lung disease	**−**	**−**	**−**	290
**c**	F	79	27	AI	Hypertension, diabetes, kidney disease	**−**	**−**	**−**	173
**d**	F	62	25	AI	Hypertension	**−**	**−**	**−**	135
**e**	M	71	21	AI	Hypertension, kidney disease	**−**	**+**	**+**	352
**f**	M	55	23	AI	Hypertension	**−**	**−**	**−**	186
**g**	M	58	N/A	Dilated CMP	Kidney disease	**−**	**−**	**−**	248
**h**	F	63	33	Intracerebral hematoma	N/A	N/A	N/A	N/A	270
**i**	M	58	N/A	CHF	TIA/Stroke, Lung disease	+	+	−	413
**j**	M	42	27	Subarachnoidal hematoma	N/A	N/A	N/A	N/A	314
**k**	F	58	59	Pulmonary embolism	TIA/Stroke	N/A	N/A	N/A	201
**l**	F	64	N/A	Dilated CMP	**-**	**−**	**−**	**−**	291
**m**	M	74	26	AMI	N/A	**−**	N/A	N/A	436
**n**	F	66	22	Epileptic seizure	N/A	N/A	N/A	N/A	347

AI indicates aortic insufficiency; CMP, cardiomyopathy; AMI, acute myocardial infarction; CHF, congestive heart failure; N/A, data not available.

### Plasma lipoproteins

Human blood plasma was obtained from healthy volunteers (Finnish Red Cross Blood Transfusion Center, Helsinki, Finland), and from the patients undergoing cardiac valve surgery. Plasma very low density lipoprotein (VLDL) (*d*<1.006 g/ml), intermediate density lipoprotein (IDL) (*d* = 1.006–1.019 g/ml), and LDL (*d* = 1.019–1.050 g/ml) were isolated by sequential ultracentrifugation in the presence of 3 mmol/l Na_2_EDTA [20], [21]. For this purpose, EDTA and 100 µg/ml Gentamicin sulfate (Lonza, Basel, Switzerland) were added to plasma, after which the plasma was centrifuged at 40 000 rpm (rotor 50.2 Ti, gmax 302 000) at +4°C for 24 h. The VLDL fraction was collected from the top of the tube, and the density of the remaining plasma was set to 1.019 g/ml with KBr. The density-adjusted plasma was then centrifuged at 40 000 rpm for 24 h, after which the IDL fraction was collected from the top of the tube. Finally, the density of the remaining plasma was set to 1.050 g/ml with KBr, the density-adjusted plasma was centrifuged at 40 000 rpm for 24 h, after which the LDL fraction was collected from the top of the tube. LDL was recentrifuged at a density of 1.063 g/ml, collected and all the lipoprotein preparations were dialyzed extensively against 1 mM EDTA - 150 mM NaCl, pH 7.4. The quantities of the lipoprotein particles are expressed in terms of their protein concentrations, which were determined by the method of Lowry et al. [22], with bovine serum albumin as standard.

### Isolation of the extracellular lipid particles

Extracellular lipid particles were isolated from the aortic valves essentially as described by Li and co-workers [23]. An entire single leaflet from each valve was used for lipid particle isolation and characterization. Briefly, each frozen leaflet was homogenized with mortar and pestle in liquid nitrogen. The homogenate was suspended in isolation buffer (0.01 M Tris - 0.15 M NaCl, pH 7.4, containing 0.02 mM butylated hydroxytoluene (BHT), 0.1% EDTA, 0.01% sodium azide (NaN_3_), and a protease inhibitor mix (Roche Complete cat 11873580001, Roche Diagnostics, Germany) in microcentrifuge tubes (LoBind, Eppendorf, Germany). First, the microcentrifuge tubes were gently shaken for 5 min, after which the tissue material was pelleted by centrifugation (10 min at 10 000 g, 4°C). The supernatants containing the extracellular lipid particles were transferred to a new tube; the pellet was resuspended in fresh isolation buffer, and placed in an ultrasonic bath in ice water to release the still remaining extracellular lipid particles. The sonicated isolate was again centrifuged (30 min at 10 000 g) to sediment the residual tissue material, and the supernatants were pooled. The final density of the lipid particle containing supernatant was 1.016 g/ml. For floating of the lipid particles by ultracentrifugation, the density of the supernatants was increased to 1.063 g/ml by adding buffer A (0.1 M Tris, 1.5 M NaCl in D_2_O; *d* = 1.116 g/ml) after which the ultracentrifuge tubes were filled with separation buffer, the density of which was set to 1.063 g/ml by mixing buffer A and buffer B (0.1 M Tris, 1.5 M NaCl in H_2_0; *d* = 1.006 g/ml). After centrifugation in a SW41Ti rotor, (Beckman Coulter, 40 000 rpm, *gmax* = 288 000× g) for 16 h at 4°C, lipid particles were recovered in 1 ml of separation buffer from the top of the ultracentrifuge tubes.

### Electron microscopy

Particle sizes were also assessed by negatively stained electron microscopy [24]. For negative staining electron microscopy, LDL and the extracellular lipid particle samples (5 µl from the isolated particles, and 5 µl from the LDL) were dried on carbon-coated grids, after which 5 µl of 1% potassium phosphotungstate, pH 7.4, was added and also dried on the grids. The samples were viewed and photographed in a JEOL 1200EX electron microscope at the Institute for Biotechnology, Department of Electron Microscopy, Helsinki, Finland.

### Fractionation of the isolated lipid particles

The lipoprotein particles were fractionated using rate zonal ultracentrifugation as described earlier [25] except that a discontinuous D_2_O density gradient was used. The gradients were created by mixing buffer A and buffer B, as described under the Methods. The buffers were layered in 9/16×3 ½ inch Ultra Clear centrifuge tubes (Beckman Coulter), coated with free fatty acid -free bovine serum albumin (Sigma) as follows: 1 ml of the solution containing the isolated extracellular lipid particles (density set to 1.094 g/ml with buffer A) was applied to the bottom of the tube. Next, three 3 ml layers of the buffers were added, their respective densities being 1.079 g/ml, 1.050 g/ml, and 1.030 g/ml. Finally, 2 ml of buffer B (*d* = 1.006 g/ml) was added. The gradients were centrifuged for 1 hour at 40 000 rpm (rotor SW-41 Ti). The layered buffer solution was then fractionated into 500 µl aliquots.

#### Analysis of the isolated lipid particles

The cholesterol content of the isolated lipid particles was measured using a fluorometric Amplex Red Cholesterol Assay Kit (Molecular Probes Europe BV, Leiden, The Netherlands). The apolipoprotein B-100 (apoB-100) -contents of the fractions were measured using specific ELISAs (MABTECH, Nacka, Sweden). The sizes of isolated lipid particles and lipoprotein particles were measured using dynamic light scattering (Zetasizer Nano, Malvern Instruments, Malvern Works, UK).

#### Determination of oxidized epitopes in LDL

Extracellular particles were isolated from 5 stenotic valve leaflets (Table 1, patients P and Q, and additional leaflets from patients K, L and M). Total valve leaflet tissue mass was 9360 mg and total protein concentration of the isolate was 0.3 mg/ml. The extracellular particles were pooled, and oxidized epitopes in the isolated particles were detected using antibodies recognizing malondialdehyde (MDA) -modified LDL (MDA-LDL) and malondialdehyde acetaldehyde (MAA) -modified LDL (MAA-LDL) epitopes [26], [27]. Monoclonal antibodies were generated by fusing mouse splenocytes with P3xAg8.653.1 myeloma cell line using standard methods, and selected based on their binding to MDA-LDL and MAA-LDL. Clones HMN-08_34 [26], HME-04_7 [27], and HME-04_6 [26] were cloned as described previously. Clone HMC+10_101 was cloned from splenocytes of C57BL/6 mouse immunized with mouse-MDA-LDL without adjuvant. ApoB containing particles and oxidized LDL-epitopes were detected by chemiluminescent immunoassay method. Samples were diluted 5 µg/ml in PBS-0.27 mM EDTA and immobilized on 96-well plate (50 µl/well) overnight at +4°C. The wells were blocked with 0.5% fish gelatin - 0.27 mM EDTA -PBS for 1 hour at room temperature. The antibodies were biotinylated and diluted in 0.5% fish gelatin - 0.27 mM EDTA - PBS as follows: HMN-08_34, 2 µg/ml, HME-04_7, 1 µg/ml, HME-04_6, 2.5 µ/ml, and HMC+10_101, 4 µg/ml. Goat anti-human apoB-48/100 (Meridian Life Sciences, Memphis, Tennessee, USA) 0.1 µg/ml was used to detect apoB containing particles. Antibody dilutions (50 µl/well) were added in duplicate wells and incubated for 1 hour at room temperature. Bound antibodies were detected with alkaline phosphatase labeled NeutrAvidin (Thermo Scientific, Rockford, Illinois, USA), and LumiPhos 530 substrate (Lumigen Co, Southfiel, Michigan, USA). NeutrAvidin-ALP was diluted 1∶18000 in 0.5% fish gelatin – 1 mM MgCl_2_- TBS and 50 µl was added in each well for 1 hour at room temperature. LumiPhos 530 was diluted 1∶3 in H_2_O and 25 µl was added in each well. After incubation for 90 minutes at room temperature, the luminescence was detected with Victor ^3^ multilabel counter. The wells were washed after each step with automated plate washer and PBS-0.27 mM EDTA. Results are expressed as relative light units measured in 100 ms (RLU/100 ms).

#### Mass spectrometry of aortic valves

For electrospray ionization-mass spectrometry (ESI-MS), the lipids in the excised aortic valves (Table 1, patients R-X) and in the extracellular particles isolated from them, were extracted [28], concentrated under nitrogen flow, and dissolved in chloroform-methanol (1∶2, v/v). The extractions were carried out twice to carefully remove any remaining water soluble substances. As control samples, lipid cores of aortic plaques obtained from abdominal aortae from autopsied subjects (n = 3), and prepared in the same way were used. The samples had been spiked with a cocktail of several quantitative standards (Table 3), and to support ionization and prevent adduct formation, just prior to the analysis, NH_3_ was added to the samples to give a final concentration of 1% (v/v). The mass spectra were recorded in positive ionization mode by using Bruker Esquire-LC ion trap ESI-MS equipment (Bruker Daltonics, Bremen, Germany). In order to resolve overlapping peak patterns and peak areas, and accurately quantify each lipid species, the data were further analyzed with DataAnalysis 3.4. (Bruker Daltonics), and LIMSA [29] software.

**Table 3:** Lipid standards used for mass spectrometry.

Lipid class	m/z	c (pmol/µl)
CE 16:0	642	7.52
SM 12:0	647	0.73
PC 40:2	842	0.29
Cer 12:0	482	0.73
LPC 14:0	468	0.73
TAG 42:0	740	1.45
PE 28:0	636	0.29
PS 28:0	676	0.29

Lipid standards used for mass spectrometric analysis of the atherosclerotic plaques, excised aortic valves and extracellular lipid particles isolated from stenotic aortic valves. Each analyzed sample was spiked with the standard mixture for quantitative measurement and the abundances of each lipid species were calculated as mole percentages.

#### Thin layer chromatography

Lipids in the different samples were extracted as described above, and the contents of neutral lipids and phospholipids were determined using thin layer chromatography (CAMAG Automatic TLC-sampler III, Mutten, Switzerland) together with a separate standard compound for each lipid class. Standard curves were also calculated separately for each lipid class. The compounds were separated on the silica plate using as eluent hexane/diethyl ether/acetic acid/water (260∶60∶4∶1, v/v/v/v) for the neutral lipids and chloroform/methanol/acetic acid/water (25∶15∶4∶1, v/v/v/v) for the phospholipids. The plates were developed by dipping them shortly in the development solution (2 M H_2_PO_4_, 0.2 M CuSO_4_) and charring them on a hot plate until all the standards representing each major lipid class of the samples became visible. The plates were subsequently scanned and measured under ultraviolet light (CAMAG TLC-scanner, Mutten, Switzerland) and the areas were integrated using CAMAG Software v, 4.06.

#### Isolation of intracellular lipid droplets from human monocyte-derived macrophage foam cells

Human monocytes were isolated from buffy coats (obtained from the Finnish Red Cross Blood Transfusion Center, Helsinki, Finland) and differentiated into macrophages in the presence of M-CSF as described in [30]. On day 7 the cells were loaded with acetyl-LDL (0.05 mg/ml) for 24 h. Acetyl-LDL was prepared by modifying human LDL by treatment with acetic acid anhydride [31]. Macrophages loaded with acetyl-LDL were washed thrice. The lipid droplets were isolated by preparing a lysate of the cells by adding 1 ml cold water to the cells, scraping them off the bottles and drawing the cells through a 26G needle several times. The cytoplasmic cholesteryl ester (CE)-containing lipid droplets were then isolated by centrifugation (24 000 rpm in a SW 41 Ti rotor at +4°C for 60 min, after which the floating fat cake, containing the lipid droplets, was collected from the top of the centrifuge tube [32].

#### Adipophilin Western blot analysis

The intracellular lipid droplets and extracellular lipid particles were delipidated [33], and analyzed for the presence of adipophilin by Western blot analysis [33], [34].

#### Statistical analysis

The statistical analysis was carried out with PASW20, using the nonparametric Kruskal-Wallis test, with the level of significance defined as *p*<0.05.

## Results

**Figure 1:**
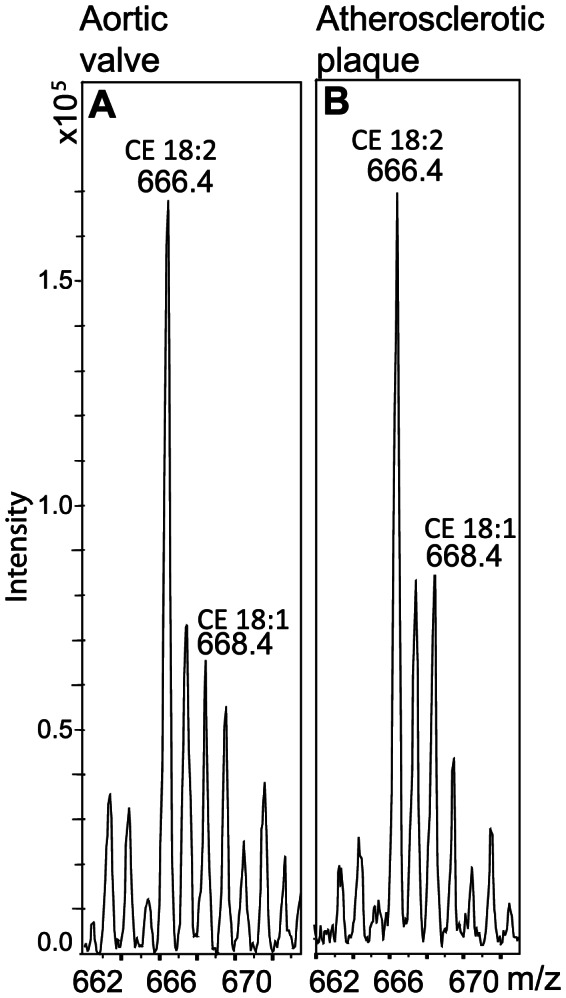
Lipid mass spectrometric analysis of a stenotic aortic valve and an atherosclerotic plaque. Lipids were extracted from a stenotic aortic valve leaflet (A) and an atherosclerotic aortic plaque (B) as described under Methods. The lipid extracts were analysed by ESI-ion trap mass spectrometer. The main CE species (cholesteryl linoleate (m/z 666) and cholesteryl oleate (m/z 668)) are indicated in the mass spectra.

**Figure 2:**
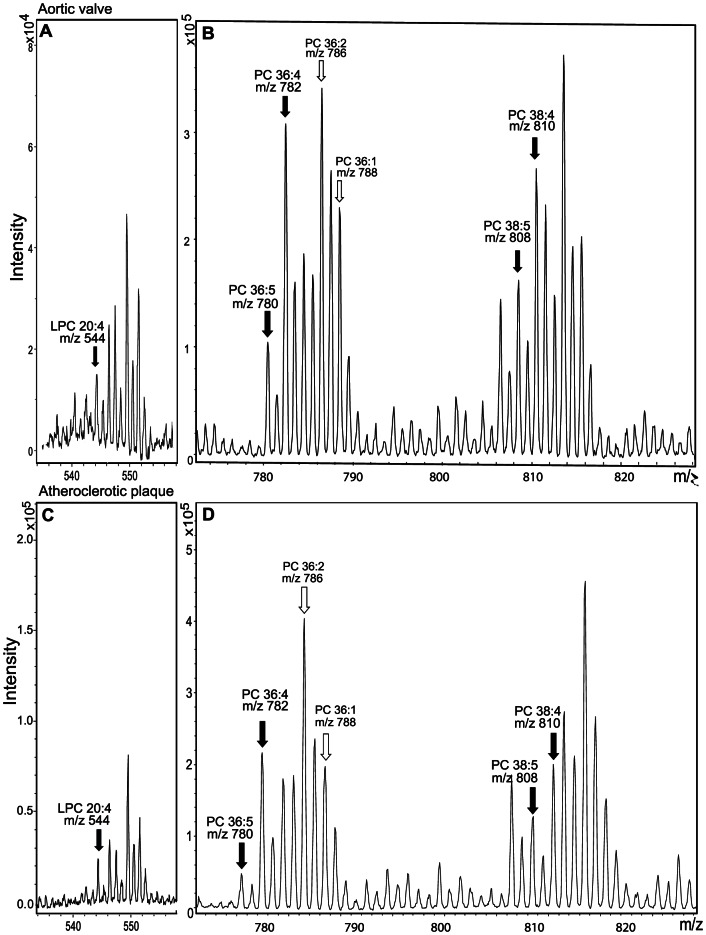
Lipid mass spectrometry analysis of PCs in a human stenotic valve and an atherosclerotic plaque. Mass spectra of LPC (Panels A and C) and PC (Panels B and D) containing arachidonic acid (20:4n-6) in human stenotic aortic valve (A and B) and human atherosclerotic plaque (C and D). The LPC species for arachidonic acid (m/z 544) in panels A and C is denoted with a black arrow. In panels B and D, the main species containing PC are 36:4 (16:0/20:4, m/z 782), 36:5 (16:1/24:4, m/z 780), 38:4 (18:0/20:4, m/z 810), and 38:5 (18:1/20:4, m/z 808) (black arrows). PC-species 36:2 (18:0/18:2, m/z 786) and 36:1 (18:0/18:1, m/z 788) (open arrows) are shown as an abundancy reference.

Lipids were extracted from 6 stenotic valve leaflets (Table 1, subjects R-X) and from the cores of 3 atherosclerotic plaques, after which their lipid compositions were analyzed by ESI-MS. Representative mass spectra are shown in Figures 1 and 2. In both cases, the major species of CEs were cholesteryl linoleate (18:2; m/z 666) and cholesteryl oleate (18:1; m/z 668). Cholesterol linoleate (18:2) is the major CE in lipoprotein particles, while cholesteryl oleate (18:1) is found particularly in the intracellular CE droplets [35]. Based on this information, the 18:1/[18:1+18:2] ratio has been used as a marker of intracellular vs. extracellular lipid accumulation [36], the ratio ranging from 0.2 to 0.47 in extracellular lipids, while being as high as 0.8 in intracellular lipid droplets. We found that in the whole stenotic aortic valves and in the atherosclerotic plaque cores, the 18:1/[18:1+18:2] ratios were, on average 0.19 (±0.05; median 0.19) and 0.22 (±0.01; median 0.23), respectively, a finding indicating that the accumulated lipids in both tissues were mainly of extracellular origin. In the healthy aortic valves, however, the amount of extracellular particles that could be isolated from a whole valve leaflet was too small to acquire reliable results of the amount of CEs or fatty acids in the extracellular lipid particles.

Atherosclerosis and aortic valve stenosis are considered inflammatory diseases. Indeed, in both tissues, large amounts of lipid species containing arachidonic acid (20:4n-6), an important inflammatory mediator [37], were seen. Thus, PC species that, according to fatty acid fragments detected, contain arachidonic acid in their sn2 position, namely 36:4 (m/z 782), 36:5 (m/z 780), 38:4 (m/z 810) and 38:5 (m/z 808), as well as arachidonic acid-containing LPC species (m/z 544), were abundant in both the atherosclerotic plaques and in the stenotic aortic valves (Figure 2). The sum of the above-mentioned PC species was, on average, 22.4% in the stenotic aortic valves and 19.1% in the atherosclerotic plaque cores (Table 4). Similarly, 12% and 6% of the LPC species in the stenotic aortic valves and in the atherosclerotic plaque cores, respectively, contained arachidonic acid.

**Table 4:** PC and LPC species extracted from atherosclerotic plaques and excised aortic valves.

			Valve	Plaque
Lipid species	Principal acyl chains	m/z	Average mol %	Average mol %
PC32:0	16:0/16:0	734	3.3±0.5	3.8±0.1
PC34:0	16:0/18:0	762	4.5±3.3	2.8±0.2
PC34:1	16:0/18:1	760	15±1.3	18±0.9
PC34:2	16:0/18:2	758	13±3.4	11±0.1
PC36:1	18:0/18:1	788	6.5±0.5	6.3±0.4
PC36:2	18:0/18:2, 18:1/18:1	786	12±1.9	12±0.7
PC36:3	18:1/18:2	784	6.2±0.7	6.0±0.7
**PC36:4**	**16:0/20:4**	**782**	**8.6±1.2**	**7.0±0.5**
**PC36:5**	**16:1/20:4**, 16:0/20:5	**780**	**3.1±0.6**	**1.8±0.4**
PC38:1	20:0/18:1	816	3.1±0.8	2.3±0.3
PC38:2	18:0/20:2, 20:0/18:2	814	6.7±1.7	7.8±0.5
PC38:3	18:0/20:3	812	4.9±0.8	6.4±0.1
**PC38:4**	**18:0/20:4**	**810**	**6.4±6.3**	**6.2±0.1**
**PC38:5**	**18:1/20:4**	**808**	**4.3±4.3**	**4.1±0.2**
PC38:6	16:0/22:6	806	3.7±0.9	5.3±0.5
LPC16:0	16:0	496	46±15	41±0.6
LPC18:0	18:0	524	30±12	32±4.9
LPC18:1	18:1	522	12±7.1	16±3.6
LPC18:2	18:2	520	12±8.7	5.1±3.4
**LPC20:4**	**20:4**	**544**	**12±4.9**	**6.1±0.9**

Extracted lipid samples were spiked with the lipid standards (Table 3), and analyzed with a mass spectrometer as described in Materials and Methods. The average abundances were expressed as mole percentages ± standard deviation. Arachidonic acid contained phospholipids are emphasized with a bolded typeface.

**Figure 3:**
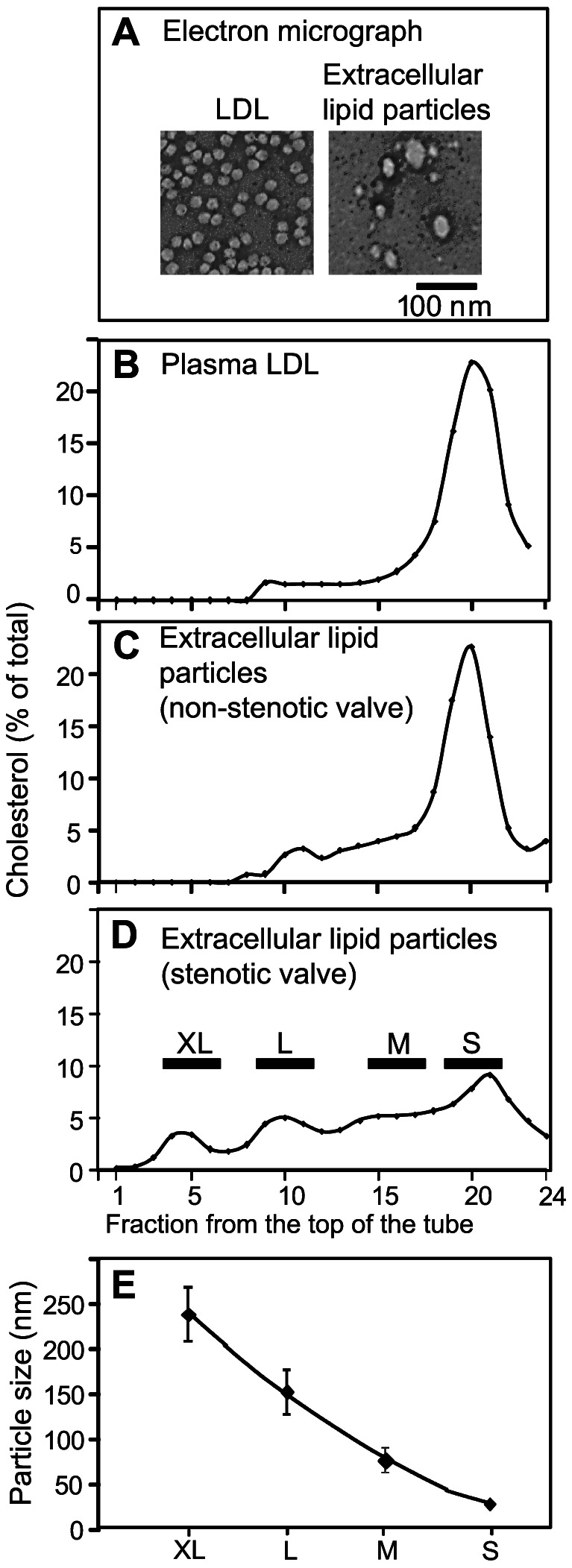
Electron micrographs and rate zonal ultracentrifugation of extracellular lipid particles. The extracellular lipid particles and native LDL were negatively stained and photographed under electron microscope as described in the Methods (A). Native LDL (B), lipid particles from a non-stenotic valve (C), and lipid particles from a stenotic valve (D) were subjected to rate zonal ultracentrifugation as described under Methods. Fractions (500 µl) were collected and their cholesterol concentrations were determined. The fractions in each sample were pooled into four groups based on the floating pattern of the extracellular lipid particles of the stenotic valves (D). The particle sizes in each pool were determined using dynamic light scattering and the average sizes of each particle class are shown in Panel E.

**Figure 4:**
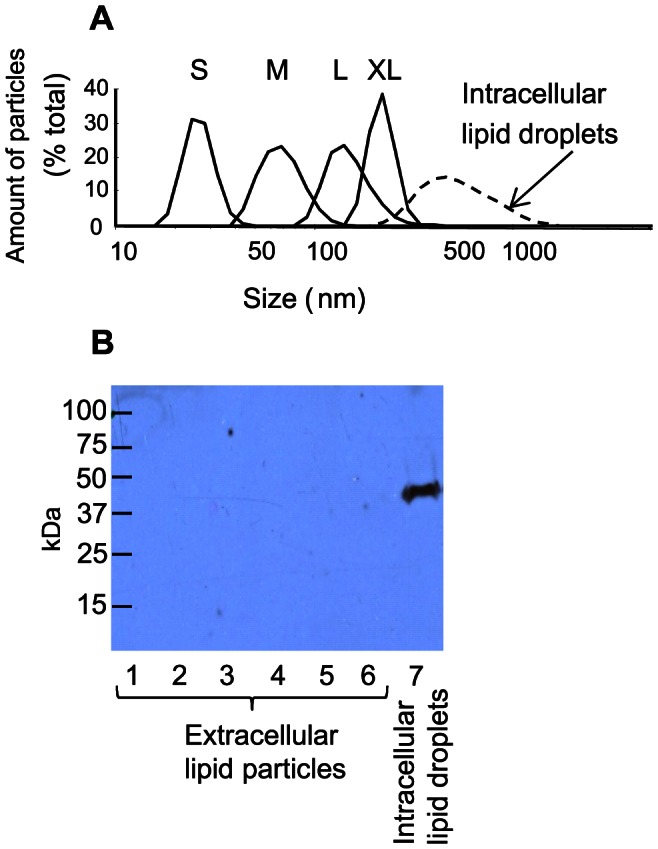
Comparison of extracellular lipid particles to intracellular lipid droplets. Intracellular lipid droplets were isolated from acetyl-LDL loaded macrophages as described in the Methods. Rate zonal ultracentrifugation of the intracellular lipid droplets was carried out, as described in the Methods. The size distributions of the intracellular particles and the extracellular lipid particle classes were measured with dynamic light scattering, extracellular particle classes (solid lines), intacellular lipid droplets (dashed line) (Panel A). The isolated lipid droplets, and extracellular lipid particles isolated from the stenotic aortic valves were analyzed by Western blot for adipophilin (Panel B) as described in Methods. Lanes 1–6: extracellular lipid particles from 6 patients; lane 7: intracellular lipid droplets from cultured monocyte-derived macrophages.

Next, we isolated lipid particles from aortic valves using a method that has been optimized for isolation of extracellular lipid particles from tissues [23]. Stenotic valve leaflets (n = 15) were much thicker and heavier than were non-stenotic leaflets (n = 14), their weights ranging from 600 mg to 2100 mg and from 130 mg to 430 mg, respectively. To isolate the lipid particles, crude isolates of the stenotic and non-stenotic valves were subjected to ultracentrifugation at a density of 1.063 g/ml, *i.e.* at a density at which VLDL, IDL, and LDL particles can be recovered from the top of the ultracentrifuge tube. After the density ultracentrifugation step, the isolated extracellular lipid particles were negatively stained and viewed under electron microscope (Figure 3 A). As the extracellular lipid particles were found to be heterogeneous in size, some particles being considerably larger than native LDL, rate zonal ultracentrifugation was used to further separate the particles into fractions according to their sizes. Figure 3 shows also representative flotation profiles of plasma LDL (Panel B), particles isolated from a non-stenotic control valve (Panel C), and particles isolated from a stenotic valve (Panel D). The flotation profile of the lipid particles isolated from the non-stenotic valve resembled that of plasma LDL, while lipid particles isolated from the stenotic valves failed to show any single major peak, but rather showed four smaller peaks, which we termed according to their sizes XL, L, M, and S. We then pooled the fractions of each peak, and the sizes of the particles in the four pools of particles in each sample were measured by dynamic light scattering (Panel E). The particles in the XL-peak had an average size of 237±30 nm (mean ± SD; fractions 4–6), those in the L-peak (fractions 9–11) 152±25 nm, those in the M-peak (fractions 15–17) 76±14 nm, and those in the S-peak (fractions 19–21) 27±1.9 nm. Representative size distributions of the particles isolated from one whole stenotic valve leaflet are shown in Figure 4A. The size distribution of the S-particles was found to resemble that of LDL-particles, while the size distribution of M-particles resembled more that of VLDL-particles. Of note, the L and XL-particles were larger than any of the apoB-100 containing particles circulating in the plasma of the patients, yet they were smaller than the experimentally created intracellular lipid droplets.

To confirm that the isolated lipid particles were mainly of extracellular origin, the particles were analyzed by Western blot for the presence of adipophilin, one of the main membrane proteins of intracellular lipid droplets [38]. Intracellular lipid droplets isolated from acetyl-LDL-loaded cultured human monocyte-derived macrophages were used as a source of intracellular CE droplets. Such droplets contained abundantly adipophilin, whereas only trace amounts of adipophilin were detected in the lipid particles isolated from stenotic valves derived from 6 patients (Figure 4B). Importantly, the intracellular lipid droplets isolated from acetyl-LDL-loaded macrophages were much larger that the lipid particles isolated from the aortic valves: the intracellular particles had an average size of 560±33 nm (Figure 4A), and they floated in fractions 1–3 of the rate zonal ultracentrifuge tubes. These findings strongly suggest that the majority of the lipids, which had accumulated in the aortic valves, were of extracellular, rather than of intracellular origin.

**Figure 5:**
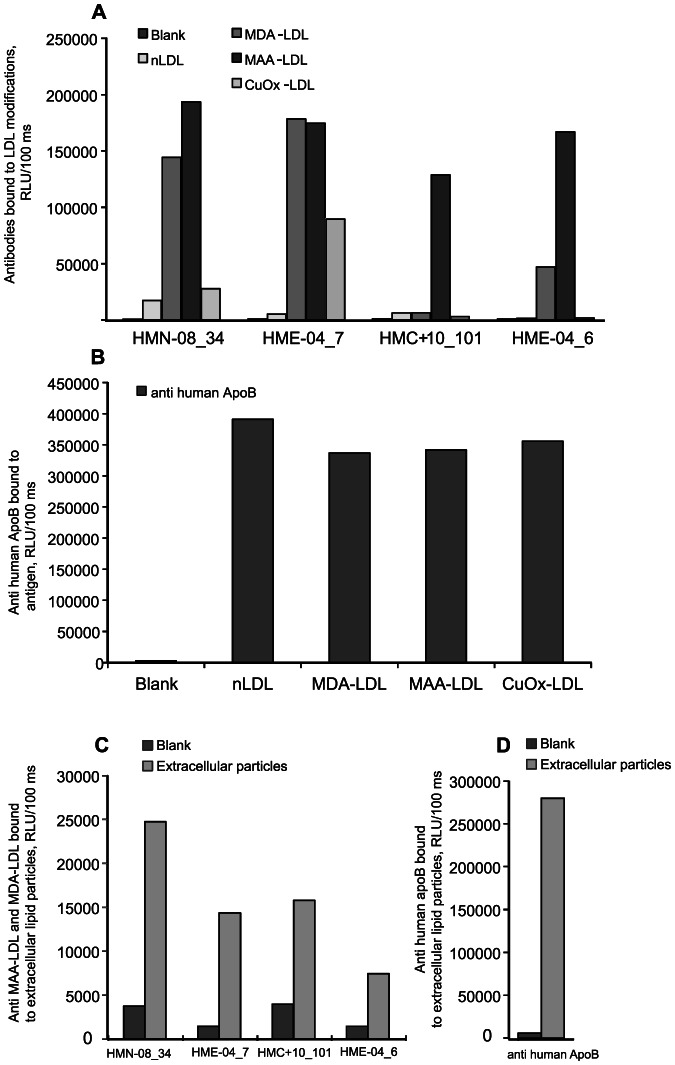
Oxidized epitopes in the extracellular lipid particles. Binding of monoclonal antibodies (clones HMN-08_34, HME-04_7, HMC+10-101 and HME-04_6) to MDA-LDL, MAA-LDL, copper-oxidized LDL (CuOx-LDL) and native LDL (A). Binding of anti-apoB antibody to the modified LDL-particles and native LDL (B). Binding of antibodies to lipid particles isolated from the stenotic aortic valves (C,D). Results are expressed as relative light units measured in 100 ms (RLU/100 ms).

To test whether the extracellular lipid particles were oxidatively modified, we examined the particles in chemiluminescent immunoassay using antibodies selected against MDA- or MAA-LDL (Figure 5). The binding of the antibodies to MDA- and MAA-modified LDL, as well as to copper oxidized and native LDL is shown in Figure 5A. Antibody clones HMN-08_34 and HME-04_7 recognized both MDA- and MAA-modified LDL, and they bound also to copper-oxidized LDL, but to a lesser extent. The clones HMC+10_101 and HME-04_6 bound mostly to MAA-modified LDL. None of the antibodies bound to native LDL. The binding of anti-human apoB antibody to MAA-LDL, MDA-LDL, copper-oxidized LDL and native is shown in Figure 5B. The extracellular lipid particles contained oxidized epitopes that were recognized by all tested MDA- and MAA-LDL binding antibodies (Figure 5C). The extracellular lipid particles were also positive for apoB, the protein component of VLDL, IDL and LDL-particles, thus revealing their origin as the apoB-100-containing plasma lipoproteins, i.e. any of the VLDL-IDL-LDL cascade.

**Figure 6:**
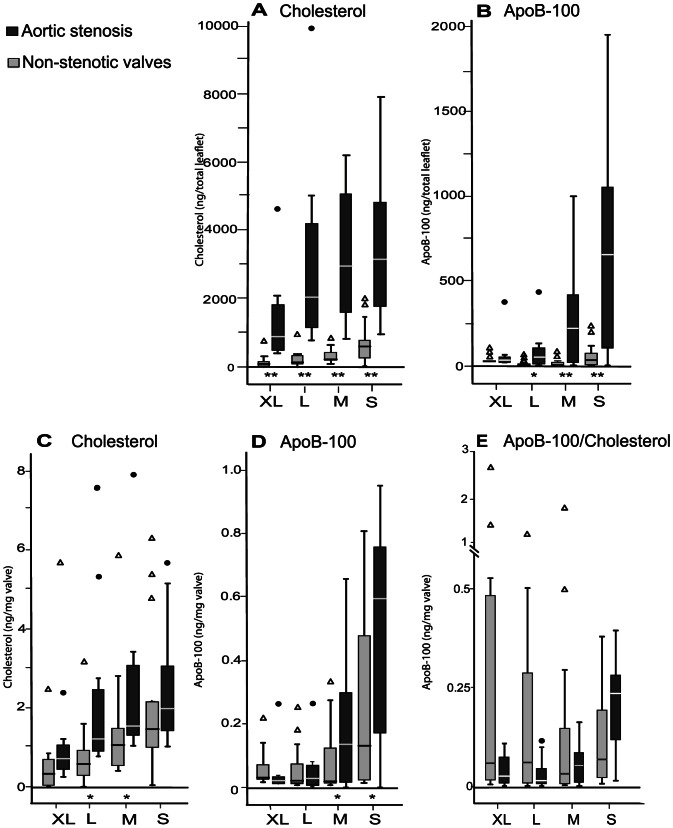
Total cholesterol and apoB-100 in extracellular particles isolated from stenotic and non-stenotic valves. Total cholesterol (A) and apoB-100 (B) in each particle class both in the stenotic and in the non-stenotic valves were measured as described in Methods. The amounts of cholesterol and apoB-100 were also calculated in proportion to the valve leaflet weight (C, D). Panel E shows the calculated relation of apoB-100 to total cholesterol in each particle class. Outliers are shown as black circles (stenotic valves) and as open triangles (non-stenotic valves). Control vs. stenotic *p<0.05, **p<0.005.

The total amounts of cholesterol and apoB-100 in the XL-, L-, M-, and S-particles isolated from the whole leaflets were next analyzed (Figures 6A and B). In each particle class, the amount of cholesterol was much higher in the particles isolated from the stenotic valves than in those isolated from the non-stenotic valves. In stenotic valves, similar amounts of cholesterol were found in the L-, M-, and S- particles, while in the non-stenotic valves, about half of the cholesterol was in S-particles, and only minor amounts were found in the larger particles. ApoB-100 was present almost exclusively in M- and S- particles isolated from the stenotic valves and in S-particles isolated from the non-stenotic valves, *i.e.* in those resembling plasma lipoproteins in their size. The amounts of cholesterol and apoB-100 were also expressed per mg wet weight leaflet tissue (Figures 6C and D). Even after the valve leaflet weight was taken into account, the amounts of cholesterol were found to be higher in particles isolated from stenotic valves than in those isolated from non-stenotic valves, the difference being statistically significant in L- and M-particle classes. In the S- and M-particles isolated from stenotic valves, the apoB-100 amounts were higher than in those isolated from non-stenotic valves, even when the valve leaflet weight was taken into account. The XL- and L-particles contained very little apoB-100 in both stenotic and non-stenotic groups.

The ratio of apoB-100 to cholesterol (Figure 6E) showed great variation among donors. In the non-stenotic valves, the proportions of cholesterol and apoB-100 were similar in all particle classes. In the stenotic valves, however, the proportion of apoB-100 was much higher in the S-particles than in the other particle classes. In all particle classes from either the stenotic or non-stenotic valves, the ratio of apoB-100 to total cholesterol was lower than in the apoB-100 containing plasma lipoproteins, in which it is, on average, 0.3 in VLDL and 0.5 in LDL [39].

**Figure 7:**
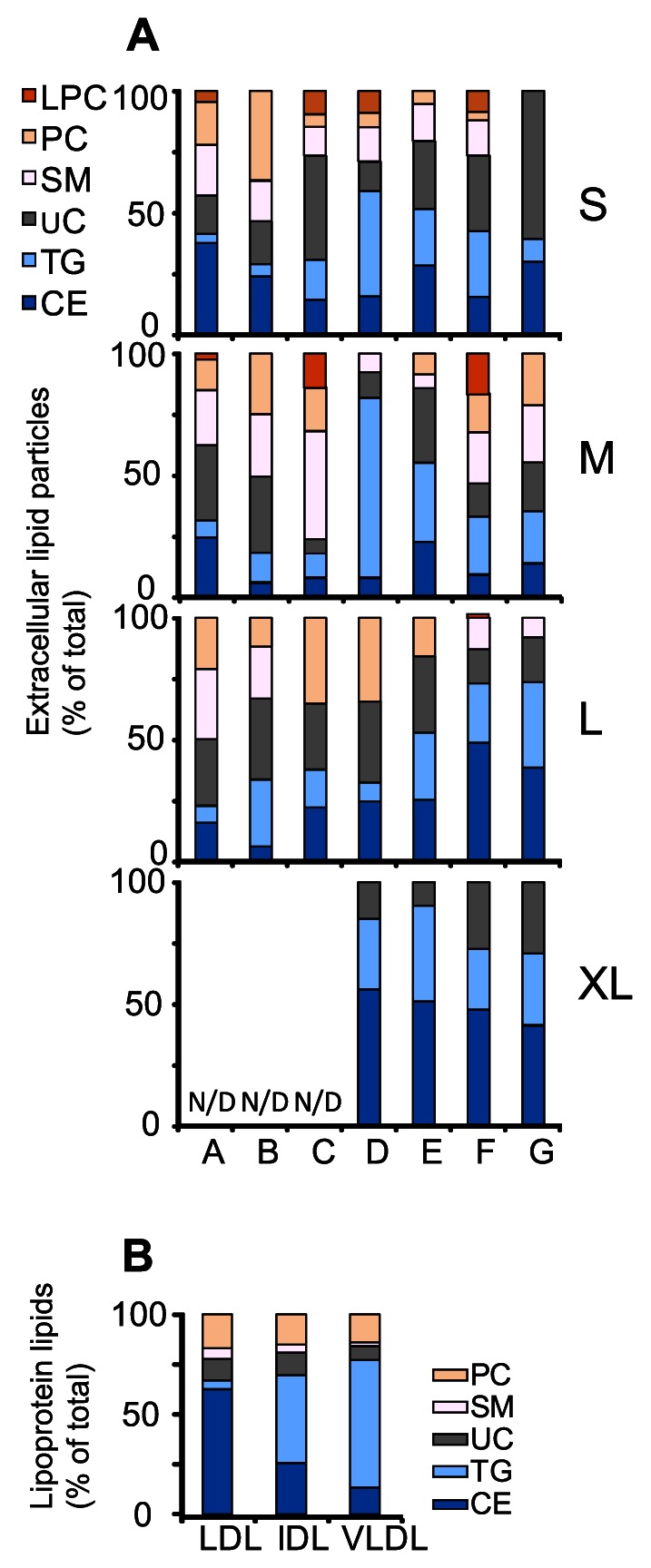
Lipid analysis of extracellular lipid particles. Lipid contents of the extracellular particles isolated from seven patients (patients A to G) were determined by TLC as described in Methods. The lipid contents of each particle class are shown as mass percentages of total lipid (A). As a comparison, the lipid contents of plasma LDL, IDL and VLDL were also determined and are shown here as mass percentages of total lipid (B).

The lipid compositions of the S-, M-, L- and XL-particles from stenotic aortic valves of seven patients was analyzed by TLC, and are shown individually as mass percentages (patients A to G in Figure 7A). For comparison, the lipid compositions of LDL, IDL, and VLDL are also shown (Figure 7B). The proportions of the lipids in the S-, M-, L- and XL- particles varied considerably among the patients. In general, the lipid profiles of the particles in the four particle classes isolated from a single donor tended to resemble each other more than the lipid profiles in a single particle class isolated from different donors. Interestingly, the lipid profiles of the particles of patient C differed from each other, the M-particles being extremely rich in phospholipids and the S- and L-particles being enriched in UC. In general, when compared to plasma lipoproteins, the ratio of PC to SM was reduced in lipid particles isolated from the stenotic valves. Also, the proportion of UC was much higher in the lipid particles than in native plasma lipoproteins, being 20–80% and 15–30% of the total cholesterol content, respectively.

## Discussion

In the present study, we have isolated and characterized extracellular lipid particles from severely stenotic and from non-stenotic human aortic valves, and we found that their average sizes ranged from 18 to 270 nm, the largest particles having diameters of about 500 nm. Earlier, the morphology of extracellular lipid droplets in the aortic valves of hypercholesterolemic rabbits has been characterized by aid of electron microscopy [18]. In remarkable consistency with the present results, the authors found that prolonged lipid feeding of rabbits was associated with the appearance of enlarged extracellular lipid particles in the aortic valves, the sizes of the particles ranging from 23 to 220 nm. In the present study, the extracellular lipid particles could be divided into four groups according to their flotation characteristics, which corresponded to their sizes. Thus, in rate zonal ultracentrifugation, four flotation maxima were observed (see Figure 4), and, accordingly, the particles were classified to S-, M-, L-, and XL-particles. The S- and M-particles were found to resemble LDL and VLDL in size, while the L- and XL- particles were larger than any of the apoB-100 -containing plasma lipoproteins, suggesting that they are formed from plasma lipoproteins by aggregation and/or fusion.

The accumulation of extracellular lipid particles in AS resembles accumulation of extracellular lipid particles in the arterial intima during atherogenesis, the morphology of which has been characterized in detail by Guyton and co-workers [36]. In atherosclerosis, the cause of lipid accumulation is considered to be the binding and entrapment of plasma lipoproteins by the components of the arterial extracellular matrix, particularly proteoglycans [40],[41],[42],[43],[44],[45]. The accumulation of apoB-100 –containing lipoproteins has also been shown in human stenotic aortic valves [12], [13], where they have been found to co-localize with biglycan and decorin [46]. Once the lipoproteins are retained by the proteoglycans in the aortic valves, they can become modified by proteases, lipases, and oxidative agents present in the extracellular fluid. Such modifications can lead to aggregation and fusion of the modified lipoproteins and enhance their binding to the extracellular matrix [47], [48], [49], [50], [51], [52], [53]. Interestingly, the extracellular lipid particles isolated from stenotic aortic valves in this study resemble such *in vitro* aggregated and fused lipoprotein particles both in size and in composition. Thus, when compared to plasma lipoproteins, the lipid particles had a decreased PC/SM-ratio, and particles from several donors also contained significant amounts of LPC. The extracellular lipid particles from most of the donors contained also very high amounts of UC. This finding is in accordance with animal studies, in which high amounts of UC were detected in the extracellular lipid particles in the aortic and atrio-ventricular valves of hypercholesterolemic rabbits [18], [54], [55].

The decreased PC/SM ratio and increased amount of LPC in the particles have likely resulted from modification of the particles by oxidation or by phospholipase A_2_ (PLA_2_), and a likely source of UC in these particles is hydrolysis of their CEs by cholesteryl esterase [56]. Indeed, the isolated lipid particles showed signs of oxidation, and oxidized LDL has been shown in stenotic aortic valves in the areas with inflammatory cell infiltration [57]. Since oxidized lipoproteins are readily taken up by macrophage scavenger receptors [58], the presence of oxidized particles in the valvular extracellular space reflects insufficient clearance of the particles by macrophages and valvular myofibroblasts. Interestingly, the amount of oxidized LDL in valves is associated with the amount of small dense LDL in circulation, *i.e.* with the oxidation-susceptible subfraction of LDL in the blood plasma [57]. Small dense LDL has been suggested to be the result of phospholipid hydrolysis on LDL particles [59] and, in fact, the plasma levels of lipoprotein-associated PLA_2_ (Lp-PLA_2_) and small dense LDL correlate [60]. Importantly, Lp-PLA_2_ is elevated in patients with severe aortic valve stenosis, and the aortic valve area correlates inversely with plasma levels of Lp-PLA_2_ [61]. Another phospholipase that may hydrolyze LDL phospholipids in the aortic valve is secreted PLA_2_ type IIA. This enzyme is present in stenotic aortic valves [62], can hydrolyze LDL particles [63] and induce their fusion [25]. UC can be generated by the action of lysosomal acid lipase, an enzyme, which has been shown to be secreted by activated macrophages [64], which are abundantly present in stenotic aortic valves [65].

The presence of oxidized LDL has been shown to be associated with the degree of valvular inflammation and tissue remodelling [2], [57]. Indeed, oxidized lipoproteins, as well as many of the products of lipoprotein modifications, such as LPC and fatty acids, are strong proinflammatory factors [66], [67], [68]. Arachidonic acid is an important mediator of inflammation [37], and, as shown in this study, arachidonic acid-containing lipid species, such as LPC and PCs are abundant in stenotic aortic valves. Another interesting bioactive molecule present in the extracellular particles is UC, which, once crystallized, may trigger a strong inflammatory response in macrophages [30], [69]. The accumulated lipid particles rich in such proinflammatory molecules can activate neighboring macrophages, and so act as a nidus for further lipid accumulation with an ensuing increase in the lipolysis-induced inflammatory burden in aortic valves. Thus, once lipoprotein particles are retained in an aortic valve, a self-perpetuating feedback loop may ensue, in which the proinflammatory lipid species generated can act as activators of the valvular cells.

The modified lipoproteins may also contribute to valvular calcification: LPC has been shown to trigger smooth muscle cells to induce calcification in lipid-rich environment [70]. The idea of lipid-induced calcification of the aortic valve is supported by the hypercholesterolemic mouse model of Miller and co-workers [71], in which hyperlipidemia resulted in elevated superoxide levels, deposition of lipids and calcium, and expression of pro-osteogenic proteins in the aortic valve. Interestingly, these adverse valvular changes, including calcification, were reversed by normalization of blood cholesterol levels.

Unfortunately, the proposal of lipid accumulation being a significant component in the development and progression of AS, as so nicely demonstrated in animal models and retrospective studies [68], has not gained support from prospective clinical data. Thus, the presumption that plasma lipid-lowering therapy is of significant therapeutic value in the prevention and treatment of AS [72], [73] has suffered severely, as the results of clinical studies involving plasma lipid-lowering therapy have been uniformly unsuccessful in slowing AS progression [5], [6]. The results of the present study involving severely stenotic aortic valves indicate that plasma lipoprotein particles have accumulated and become extensively modified in the extracellular space of the diseased valves. Based on this finding, we propose that the type and extent of lipoprotein modification in the valves, rather than high levels of circulating plasma lipoproteins *per se*, are responsible for the initiation and/or progression of AS. Since in the diseased aortic valves, denudation of the endothelial cell layer can be observed, the endothelial barrier function mechanism may be lost, so allowing free access of LDL particles to the subendothelial extracellular matrix, irrespective of their concentrations in the plasma [74]. An important corollary of this proposition is that, even during statin treatment, the plasma levels of apoB-containing lipoproteins are high enough to allow lipid accumulation to proceed. Therefore, as the stenosis develops slowly in the course of many years, even low infiltration rates of the particles appear to be sufficient to allow a continuous particle supply for the local modifying processes. Thus, in addition to plasma lipid lowering strategies, we should also aim at achieving resolution of the ongoing inflammatory activation in the diseased valves. Since some of the promising novel anti-inflammatory mediators are derivatives of the polyunsaturated fatty acids, including arachidonic acid [75], we face a difficult, but hopefully an ultimately rewarding challenge of resolving the lipid-dependent proinflammatory state in the valves and switching it into a lipid-dependent anti-inflammatory state.
